# Alzheimer’s Disease: An Update and Insights Into Pathophysiology

**DOI:** 10.3389/fnagi.2022.742408

**Published:** 2022-03-30

**Authors:** Murtala Bello Abubakar, Kamaldeen Olalekan Sanusi, Azizah Ugusman, Wael Mohamed, Haziq Kamal, Nurul Husna Ibrahim, Ching Soong Khoo, Jaya Kumar

**Affiliations:** ^1^Department of Physiology, Faculty of Basic Medical Sciences, College of Health Sciences, Usmanu Danfodiyo University, Sokoto, Nigeria; ^2^Centre for Advanced Medical Research and Training, Usmanu Danfodiyo University, Sokoto, Nigeria; ^3^Department of Physiology, Faculty of Medicine, Universiti Kebangsaan Malaysia Medical Centre, Kuala Lumpur, Malaysia; ^4^Department of Basic Medical Science, Kulliyyah of Medicine, International Islamic University Malaysia, Kuantan, Malaysia; ^5^Department of Clinical Pharmacology, Faculty of Medicine, Menoufia University, Shebin El-Kom, Egypt; ^6^Neurology Unit, Department of Medicine, Faculty of Medicine, Universiti Kebangsaan Malaysia Medical Centre, Kuala Lumpur, Malaysia

**Keywords:** Alzheimer, diagnose, therapeutic, JAK, NGF, omic, dementia, vascular

## Abstract

Alzheimer’s disease (AD) is an irreversible brain disorder associated with slow, progressive loss of brain functions mostly in older people. The disease processes start years before the symptoms are manifested at which point most therapies may not be as effective. In the hippocampus, the key proteins involved in the JAK2/STAT3 signaling pathway, such as p-JAK2-Tyr1007 and p-STAT3-Tyr705 were found to be elevated in various models of AD. In addition to neurons, glial cells such as astrocytes also play a crucial role in the progression of AD. Without having a significant effect on tau and amyloid pathologies, the JAK2/STAT3 pathway in reactive astrocytes exhibits a behavioral impact in the experimental models of AD. Cholinergic atrophy in AD has been traced to a trophic failure in the NGF metabolic pathway, which is essential for the survival and maintenance of basal forebrain cholinergic neurons (BFCN). In AD, there is an alteration in the conversion of the proNGF to mature NGF (mNGF), in addition to an increase in degradation of the biologically active mNGF. Thus, the application of exogenous mNGF in experimental studies was shown to improve the recovery of atrophic BFCN. Furthermore, it is now coming to light that the FGF7/FGFR2/PI3K/Akt signaling pathway mediated by microRNA-107 is also involved in AD pathogenesis. Vascular dysfunction has long been associated with cognitive decline and increased risk of AD. Vascular risk factors are associated with higher tau and cerebral beta-amyloid (Aβ) burden, while synergistically acting with Aβ to induce cognitive decline. The apolipoprotein E4 polymorphism is not just one of the vascular risk factors, but also the most prevalent genetic risk factor of AD. More recently, the research focus on AD shifted toward metabolisms of various neurotransmitters, major and minor nutrients, thus giving rise to metabolomics, the most important “omics” tool for the diagnosis and prognosis of neurodegenerative diseases based on an individual’s metabolome. This review will therefore proffer a better understanding of novel signaling pathways associated with neural and glial mechanisms involved in AD, elaborate potential links between vascular dysfunction and AD, and recent developments in “omics”-based biomarkers in AD.

## Introduction

Alzheimer’s disease (AD) is a progressive brain disease that is attributed by the Alzheimer Association as the cause of 60–80% of dementia cases. Depending on the stage of the disease, it is characterized by factors that progress to hinder the performance of everyday activities, such as apathy, depression, impaired communication, disorientation, poor judgment, difficulty in swallowing and walking, and behavioral changes (Alzheimer’s Association, 2021). The duration takes to develop a continuum of these symptoms is determined by factors such as age, genetics, and sex (Vermunt et al., 2019). Current statistics show that over six million Americans aged 65 and above are living with AD, with a projection of about 13.8 million by 2060, and death cases have increased by 16% during the COVID-19 pandemic (Alzheimer’s Association, 2021). Aside from informal caregiving, the total cost of care payment for AD patients and related diseases was estimated at $355 billion in 2021.

The progressive cognitive decline in AD is associated with the accumulation of amyloid-beta (Aβ) and tau proteins (Selkoe and Hardy, 2016). Aβ is derived from the sequential cleavage of amyloid precursor protein (APP) by beta-secretase and gamma-secretase. The aggregation of Aβ thus forms oligomers that are toxic to the neurons (Haass and Selkoe, 2007). Tau, on the other hand, is derived from alternative splicing from the microtubule-associated protein tau (MAPT) gene to form soluble protein isoforms (Goedert et al., 1989). Several functional interactions have been revealed between Aβ and tau in the neural circuit damage and cognitive decline in AD (Tripathi and Kalita, 2019; Busche and Hyman, 2020). This is in a bid to encourage a broad approach in the design of a potential therapy.

Unfortunately, no treatment option is available to cure the disease to date (Husna Ibrahim et al., 2020). Recent approaches in the treatment of AD include exploring the potentials of some natural products with neuroprotective effects (Kamil et al., 2018, 2020; Prom-In et al., 2020; Kamal et al., 2021) and metabolites to modulate signaling pathways associated with neurovascular endothelium through multi-omic analyses (Corral-Jara et al., 2021). There have also been reports on cellular signaling-related sex-dependent effects under hyperglycemic and lipid stress (Nuthikattu et al., 2020, 2021). However, this review focuses on significant signaling pathways associated with the stages of AD, the potential links between vascular dysfunction and AD, as well as recent developments in “omics”-based approaches in AD. Without publication date restriction until May 2021, PubMed, ScienceDirect, and Google Scholar databases were searched for published articles containing the search terms related to “Alzheimer,” “dementia,” “signaling pathways,” “vascular dysfunction,” “cognitive impairment,” and “omics.” Relevant articles retrieved were thereafter included for synthesis.

## Signaling Pathways Associated With Alzheimer’s Disease

From several studies, a group of researchers was able to develop a database containing a compilation of signaling pathways associated with AD, called AlzPathway^1^ (Mizuno et al., 2012). There are several components of the AlzPathway, which include the Aβ cleavage and degradation, apolipoprotein E (ApoE)-cholesterol pathway and NFT accumulation, acetylcholine production, Wnt signaling pathway, Ubiquitin mediated proteolysis, apoptosis, calcium signaling pathway, Notch signaling pathway, MAPK signaling pathway, abnormal ceramide accumulation, reactive oxidation process, neurotrophin signaling pathway, cell cycle, mTOR signaling pathway, lipid pathway, insulin pathway, and inflammation pathway (Mizuno et al., 2012). Here, we discuss newly emerging pathways as potential diagnostic and therapeutic targets.

In the 2021 Alzheimer Drug Development Pipeline, agents that are currently in clinical trials were divided into 3 categories; agents that are disease-modifying biologic, disease-modifying small molecule, and symptom-reducing small molecule (Cummings et al., 2021). Twenty-nine percentage of the disease-modifying therapies (DMTs) that were successfully shifted to the 3rd phase trials are targeting the amyloid. Meanwhile, out of 50 drugs that were repurposed for AD treatments, 10 of the agents proceed to Phase 3 clinical trials. AD drug development has been very challenging due to its heterogeneity in AD pathogenesis, with a 0.4% success rate as memantine is the only drug that has significant effects and was approved by FDA out of 244 drugs investigated in AD clinical trials from the year 2002 to 2012 (Fish et al., 2019).

As microglia activation also plays a role in the pathogenesis of AD, minocycline, an anti-inflammatory tetracycline drug was investigated in AD clinical trials due to its ability to penetrate the blood-brain barrier (BBB) while hindering the proinflammatory microglia. Besides, minocycline attenuates the fibrillization of Aβ, hence, halting the deposition of Aβ plagues and neuronal death *in vitro* (Romero-Miguel et al., 2021). However, 24 months of 400 mg minocycline treatment in a randomized clinical trial failed to delay the cognitive impairments in mild AD patients (Howard et al., 2020). This finding is parallel to the previous trials on non-steroidal anti-inflammatory drugs (NSAIDs) which failed to exert positive effects on delaying AD developments. Thus, it can be deduced that maybe although neuroinflammation is one of many pathways involved in the heterogenic pathogenesis of AD, targeting neuroinflammation alone may not be enough to exert significant neuroprotective effects in AD.

Vitamin E is an antioxidant consisting of α-, β-, γ-, and δ-tocophenols and tocotrienols, that can neutralize free radicals and ROS which eventually protecting the cellular membrane from the destructive oxidative stress (Lloret et al., 2019). α-tocopherol is a critical antioxidant in the brain due to the abundance α-tocopherol transfer protein (α-TTP) transporter for the regulation of vitamin E distribution in other tissues. Besides, vitamin E is also involved in the protein kinase C (PKC) pathway and exerts anti-inflammatory effects. The first clinical trial of vitamin E in 1997 showed a decline in progression of AD which remarkably shifted the attention toward the advancement of vitamin E studies in the development of AD treatments. However, several failed clinical trials later left the efficacy of vitamin E questionable. The study results may have been influenced by the discrepancies in the bioavailability of vitamin E such as the base nutritional status, intestinal differences, and rate of absorption for each cohort in the clinical trials, alongside the acclaimed heterogeneity of AD pathogenesis itself (Table 1).

**TABLE 1:** Agents in Phase 3 clinical trials from 2016 to 2021.

2016	2017	2018	2019	2020	2021
Albumin + IVIG CAD106 Gantenerumab Solanezumab Aducanumab	Albumin + IVIG CAD106 Gantenerumab Solanezumab Aducanumab Crenezumab	Albumin + IVIG CAD106 Gantenerumab Solanezumab Aducanumab Crenezumab	Plasma exchange with Albumin + IVIG CAD106 Gantenerumab Solanezumab Aducanumab Crenezumab	CAD106 Gantenerumab Solanezumab Aducanumab BAN2401	Gantenerumab Solanezumab Aducanumab Lecanemab
MK-8931 Pioglitazone CNP520 GV-971 ALZT-OP1a/b Nilvadipine Insulin AZD3293 TTP488 JNJ54861911 TRx0237 Masitinib	MK-8931 Pioglitazone CNP520 GV-971 ALZT-OP1a/b Nilvadipine Insulin AZD3293 JNJ54861911 TRx0237 Azeliragon E2609	MK-8931 CNP520 GV-971 ALZT-OP1a/b Insulin AZD3293 JNJ54861911 TRx0237 Azeliragon E2609 Icosapentethyl (IPE)	CNP520 ALZT-OP1a/b TRx0237 E2609 COR388 ANAVEX2-73 Icosapentethyl (IPE) Losartan + Amlodipine + Atorvastatin AGB101 BHV4157 Masitinib	ALZT-OP1a/b TRx0237 COR388 ANAVEX2-73 Icosapentethyl (IPE) Losartan + Amlodipine + Atorvastatin AGB101 BHV4157 Masitinib Azeliragon Metformin Tricaprilin	TRx0237 Icosapentethyl (IPE) Losartan + Amlodipine + Atorvastatin AGB101 Atuzaginstat Azeliragon Blarcamesine GV-971 Metformin NE3107 Tricaprilin Troriluzole Omega-3
AC-1204 Aripiprazole AVP-786 Brexpiprazole Lu AE58054 Nabilone RVT-101	AC-1204 Aripiprazole AVP-786 Brexpiprazole Nabilone RVT-101 ITI-007 Methylphenidate Idalopirdine Suvorexant	AVP-786 Nabilone ITI-007 Methylphenidate Suvorexant AXS-05 Escitalopram Octohydroaminoacridine succinate Zolpidem	AVP-786 Brexpiprazole Nabilone Methylphenidate AXS-05 Mirtazapine Escitalopram Octohydroaminoacridine succinate Guanfacine Zolpidem Ginkgo Biloba	AVP-786 Brexpiprazole Methylphenidate AXS-05 Mirtazapine Escitalopram Octohydroaminoacridine succinate Guanfacine Zolpidem Ginkgo Biloba BPDO-1603 Zoplicone	AVP-786 Brexpiprazole Nabilone Mirtazapine Escitalopram Octohydroaminoacridine succinate Guanfacine Ginkgo Biloba BPDO-1603 Caffeine Donepezil

*Agents in Phase 3 since 2016–2021 (Cummings et al., 2016, 2017, 2018, 2019, 2020, 2021).*

### The Neural Mechanisms Involved in Alzheimer’s Disease

Currently, the major theories related to the mechanisms involved in the pathogenesis of AD are the neuronal extracellular deposition of Aβ peptides (senile/amyloid plaques), and neuronal intracellular accumulation of hyperphosphorylated tau protein to form neurofibrillary tangles (NFTs). However, the major underlying factor for the cognitive and behavioral dysfunction observed in AD is synaptic dysfunction. For instance, human neuronal dysfunction is associated with increased Aβ and phosphorylated tau reduces synaptic strength due to its aggregation in the dendritic spine and consequent internalization of N-methyl-d-aspartic acid receptors (NMDARs) (Sperling et al., 2009; Palop and Mucke, 2010; Wesson et al., 2011). Other factors such as oxidative stress, which is increased in the brain in aging, have been shown to precede the formation of senile plaques and deposition of NFTs (Huang et al., 2016; Singh et al., 2016; Uddin and Kabir, 2019). Moreover, increased levels of inflammatory cytokines and associated genes have also been implicated in the development of AD (Hollingworth et al., 2011; López González et al., 2016). Some emerging neural mechanistic signaling pathways that have been associated with the pathogenesis of AD are discussed below.

#### The Nerve Growth Factor Metabolic Pathway

Nerve growth factor (NGF) is a member of the neurotrophin family, which plays a critical role in the functions of the central and peripheral nervous systems (PNSs). NGF was earlier recognized for its notable role in the development of the embryo, the differentiation and survival of the PNS, as well as the differentiation and maintenance of the PNS in adulthood (Levi-Montalcini and Angeletti, 1968; Levi-Montalcini, 1987). The role of NGF in the central nervous system (CNS) was later recognized from the expression of NGF receptors in the basal forebrain cholinergic neurons (BFCN) (Schwab et al., 1979; Seiler and Schwab, 1984).

NGF is mainly released as a precursor protein (proNGF) into the extracellular space, which is then cleaved by the action of a serine protease, plasmin, to mature NGF (mNGF) (Bruno and Cuello, 2006). Upon the release of proNGF by the postsynaptic neurons of the cortex and hippocampus, and its conversion to mNGF, the physiological roles of NGF are executed by binding to two specific cell-surface receptors, the tropomyosin-related kinase A (TrkA) receptor and p75 neurotrophin receptor (p75NTR). When bound to its receptor, predominantly TrkA, on pre-synaptic cholinergic neurons, NGF gets to the cell bodies of the innervating neurons, the BFCN, through a retrograde transport, to initiate, a signaling cascade for the release of acetylcholine. NGF maturation stimulated by plasmin is positively regulated by tissue plasminogen activator (tPA) and urokinase plasminogen activator (uPA), which convert plasminogen to plasmin, and are negatively regulated by plasminogen activator inhibitor 1 (PAI-1) and neuroserpin, inhibiting the activators. The mNGF degradation, on the other hand, is by activated matrix metalloproteinases (MMPs), for example, MMP-9 release upon neuronal stimulation, and regulated by the tissue inhibitor of metalloproteinases 1 (TIMP-1). Plasmin is as well responsible for the activation of proMMP-9 to MMP-9. In addition, it has also been shown that there is *in-vitro* and *in-vivo* degradation of mNGF by MMP-3, a protease produced in response to cholinergic stimulation in cortical cells. Although alteration in MMP-3 has been shown to be sex-specific, its level is associated with cognitive decline and AD pathology in humans in addition to its observed upregulation in the experimental model of AD (Pentz et al., 2021).

In AD, this coordinated array of activities is altered at different levels of the pathway. These include alteration in the conversion of proNGF to mNGF, downregulation of TrkA receptor, impaired retrograde signaling and transport, increased mNGF degradation, and inflammatory response due to reduced acetylcholine production and increased Aβ levels (Mitra et al., 2019). Therefore, the NGF metabolic pathways involve a coordinated activity of proteases responsible for NGF maturation (e.g., tPA, plasmin, and neuroserpin) as well as those involved in NGF degradation (e.g., MMP-3, MMP-9, and TIMP-1).

Owing to the promising effect of NGF in ameliorating pathologic conditions associated with AD, a number of approaches have been made and ongoing to therapeutically deliver NGF to the diseased brain (Mitra et al., 2019). Studies in experimental model organisms have shown that the t-PA/plasmin system is an important factor that hinders the pathogenesis of AD, through its involvement in the clearance of Aβ microaggregates as well as inhibition of Aβ-induced neurodegeneration (Melchor et al., 2003; Oh et al., 2014). However, human cohort studies showed no significant alteration in the net enzymatic activities of plasmin in AD, although there was an observed increase in the mRNA level of both activators (tPA and uPA) and inhibitors (PAI-1 and α2-antiplasmin) of the plasminogen system, with increases more at the late Braak stage (Barker et al., 2012). Studies have reported increased levels of MMPs such as MMP-1, MMP-2, MMP-3, MMP-9, MMP-13, and MT1-MMP, in AD, as well as their neuroprotective effect and Aβ cleavage properties in the animal model of AD (Fragkouli et al., 2014; Kaminari et al., 2017). As beneficial as some of these proteins appear to be in AD, efforts to overexpress them have been reported to be detrimental (Wilcock et al., 2011; Terni and Ferrer, 2015), thus caution has to be taken in any chosen therapeutic approach.

#### The Janus Kinase/Signal Transducer and Activator of the Transcription Pathway

The Janus kinase/signal transducer and activator of the transcription (JAK/STAT) pathways ensure that an extracellular signal is translated into a transcriptional response through a direct mechanism.

The JAK/STAT signaling is a pathway responsible for the regulation of the cytokine responsive genes by transducing extracellular signals from ligands such as cytokines, hormones, and growth factors to the nucleus. This pathway is known to regulate processes such as cell growth and proliferation, differentiation, and apoptosis. The binding of these ligands to their receptors activates JAKs, which are of four family members: JAK1, JAK2, JAK3, and Tyk2 (tyrosine kinase 2). This will in turn phosphorylate the receptors as well as the JAKs. The STAT proteins, consisting of seven members: STAT1, STAT2, STAT3, STAT4, STAT5A, STAT5B, and STAT6, are therefore recruited to the sites formed by the phosphorylated JAKs. The STATs became phosphorylated and activated to form dimers. The dimerized STATs translocate to the nucleus to bind specific regulatory sequences and regulate gene expression (Kisseleva et al., 2002; Rawlings et al., 2004).

Evidence linking the involvement of oxidative stress to the pathogenesis of AD is buttressed by the role of reactive oxygen species in the activation of the JAK/STAT signaling pathway (Simon et al., 1998). Moreover, inhibition of the JAK/STAT pathway by various pharmacological agents has been shown to have a protective effect on AD, through the regulation of Nrf2 signaling (Sharma et al., 2020). In addition, the potential therapeutic role of receptor activation (e.g., TREM2) and folic acid supplementation has been associated with the modulation of the JAK/STAT signaling pathway (Li et al., 2016; Ruganzu et al., 2021). Micropeptides such as humanin and colivelin have also been shown to have neuroprotective effects in models of AD by activating the JAK2/STAT3 signaling pathway (Chiba et al., 2009). Moreover, a combinatorial study involving data from genetic, experimental, observational, and empirical analyses further emphasized the importance of the JAK-STAT signaling as a potential therapeutic target for AD (Nevado-Holgado et al., 2019).

Various experimental models have shown that phosphorylated STAT co-localizes with markers of astrocytes and microglia but not neurons. In addition, some genes such as Ntrk3, Cox17, and Grid2ip have been identified as the cell-specific target, because their predominant expression in astrocytes points to a specific role of STAT3 in AD pathology (Chintapaludi et al., 2020). One of the several functions of the astrocyte in the brain is the regulation of synaptic transmission, and protection and support for neurons, through the release of growth factors and cytokines (Murphy, 1993; Pascual et al., 2005). Astrocytes have been shown to play a crucial role in the degradation of amyloid plaques through the production of TGF-β to inhibit microglial activity, as well as the modulatory effect of ApoE, which is highly expressed in astrocytes (Vincent et al., 1997; Wegiel et al., 2000; Wyss-Coray et al., 2003; Koistinaho et al., 2004). Therefore, loss of astrocyte function has been found central to the pathogenesis of AD (Acosta et al., 2017). In AD, there is astrocyte involvement in oxidative stress, suppression of innate immunity, and increased expression of pro-inflammatory cytokines, suggesting the relevance of astrocyte-targeted anti-inflammatory therapy (Furman et al., 2012; González-Reyes et al., 2017; Kamal et al., 2020).

Increased expression of proteins such as glial fibrillary acidic protein (GFAP) was recognized as a “gold standard” biomarker of reactive astrocyte, a responsive state of astrocyte to a pathology (Eng et al., 2000). GFAP expression was shown to increase in the Braak stage (Simpson et al., 2010). Other potential protein biomarkers not entirely astrocyte-specific but have been shown to be altered in AD-related astrocyte dysfunction include excitatory amino acid transporters (EAATs) (of which EAAT2 has been shown to decrease with an increase in Braak stage) (Simpson et al., 2010), astrocyte-derived S100B, CD44, glutamine synthetase, and aldehyde dehydrogenase 1 family, member L1, (ALDH1L1) (Garwood et al., 2017).

#### The FGF7/FRFR2/PI3K/AKT Pathway

Fibroblast growth factors (FGFs) FGF family of growth factors is a large family of proteins with 22 identified ligands. They bind to high-affinity tyrosine kinase receptors, the FGFRs, to exert their cellular and physiological effects, which include proliferation, angiogenesis, invasion, and migration. This binding activates the intracellular tyrosine kinase domain of FGFR by phosphorylation of specific tyrosine residues. Once activated, the FGFR couple downstream intracellular signaling pathways, one of which is the PI3K/AKT signaling. In the PI3K/AKT pathway, the protein GRB2 is recruited, which then recruits another protein GAB1 to activate PI3K. Activated PI3K thereafter phosphorylates AKT (Beenken and Mohammadi, 2009; Ornitz and Itoh, 2015).

**FIGURE 1:**
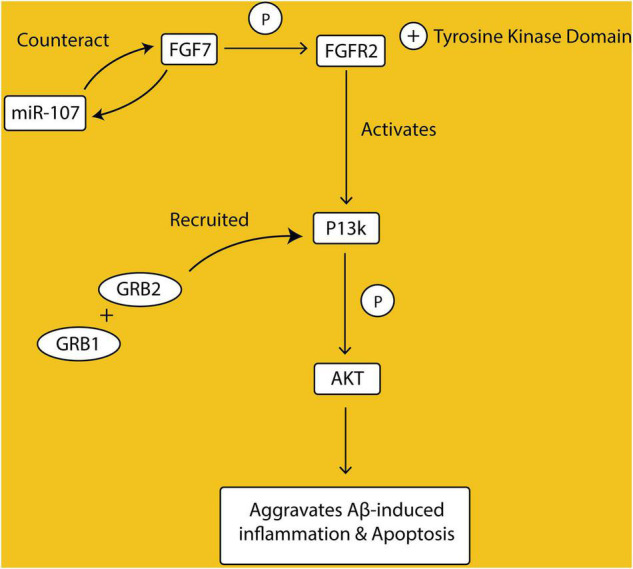
Fibroblast growth factors (FGFs) bind to their receptors (FGFRs), activating their tyrosine kinase domain through phosphorylation. FGF/FGFR complex initiates the downstream signaling, by recruiting Growth factor receptor-bound protein 2 (GRB2) and Growth factor receptor-bound protein 1 (GRB1), which then activate Phosphoinositide 3-kinases (P13K) and phosphorylate Protein kinase B (AKT). Activation of P13K/AKT pathway exacerbates amyloid beta-induced inflammation and apoptosis. FGF7 has a binding site on MicroRna17 (miR-107) and overexpression of miR-107 reduces FGF7 levels.

FGF/FGFR signaling is involved in the development of many organs in the body, including the brain, and its deregulation is associated with a variety of disease conditions (Xie et al., 2020). Among the family of FGFs, the mRNA levels of FGF7 have been shown to be elevated in patients with AD and cells treated with β-amyloid peptides (Aβ). In corroboration, overexpression of FGF7 in Aβ-treated cells was also shown to be associated with a reduction in cell viability and proliferative rate. Studies have indicated altered expression of miRNAs in the pathogenesis of AD and in experimental models of AD (Samadian et al., 2021). There is reduced expression of miR-107 in the cell model of AD and in CSF samples from AD patients (Chen et al., 2020). Interestingly, the 3′UTR of FGF7 was identified to have its binding site on the miR-107. Overexpression of miR-107 in AD model cells treated with Aβ reduced mRNA and protein levels of FGF7, and as well reduced inflammation and apoptosis, and restored cell viability (Chen et al., 2020). Western blotting analysis showed detection of FGFR2, PI3K, Akt, and Akt phosphorylation as a downstream pathway of FGF7. Therefore, data from this study implicates the involvement of the FGF7/FGFR2/PI3K/Akt signaling pathway in the pathogenesis of AD and hence a potential for therapeutic intervention (Figure 1).

Some important proteins and their modulating factors associated with the above AD pathways are shown in Table 2.

**TABLE 2:** Important proteins involved in the AD pathways.

Protein	Model	Relationship with AD pathology	Protein (or gene) changes in AD in pre-clinical and clinical stages	Modulating drug	Drug effect	Drug mechanism of action	References
**NGF metabolic pathway**							
tPA/plasmin system	APPswe/PS1 transgenic mice	Cerebral Aβ levels and cognitive function		Recombinant tPA (Activase rt-PA)	Reduced cerebral Aβ levels and improved cognitive function	Increased frequency of monocytes in circulation and brain microglia stimulation for neuroprotective phenotype	ElAli et al., 2016
				TM5275 (inhibitor of PAI-1)	Hippocampal and cortical reduction of Aβ load, and improvement of learning/memory function	Increased activities of tPA, uPA and plasmin, and LRP-1-mediated efflux of Aβ in the brain	Akhter et al., 2018
				Tert-butyl hydroquinone	Reduction of Aβ load and oxidative stress	Inhibition of PAI-1, stimulation of Aβ degradation, and increased antioxidant capacity.	(Akhter et al., 2011)
	Human	Activated by Aβ	Increase in the mRNA level of both activators (tPA and uPA) and inhibitors (PAI-1 and α2-antiplasmin) at late Braak stage				Barker et al., 2012
MMPs	Human	Microglial activation and inflammation	Increase in preclinical (e.g., MMP-3) and clinical (e.g., MMP-3) stages of AD				Hanzel et al., 2014; Pentz et al., 2021
**JAK/STAT pathway**							
p-JAK2-Tyr1007 and p-STAT3-Tyr705	APP/PS1 transgenic mice	Neuroinflammation	Increased level in AD mice	Suan-Zao-Ren Decoction	Anti-inflammatory action	Downregulation of hippocampal JAK2/STAT3-related signaling pathway.	Long et al., 2021
p-JAK2 and p-STAT3	Aβ1–42-induced AD mice model	Neuroinflammation	Downregulation in AD	Hydroxy-safflor yellow A	Inhibited inflammatory response	Up-regulated the JAK2/STAT3 pathway and inhibited the activation of NF-κB signaling pathways.	Zhang et al., 2014
p-JAK2-Tyr1007/1008 and p-STAT3-Tyr705	N2a Swe cells	Increased Aβ and C99 levels	Increased in activated N2a Swe cells	Taxifolin and cilostazol	Inhibited amyloidogenesis	Suppressed P- JAK2/STAT3/NF-κB- signaling *via* up-regulation of SIRT1	Park et al., 2016
**FGF7/FGFR2 pathway**							
FGF7	Aβ-induced SH-SY5Y cells	Upregulation promotes inflammation and apoptosis	Elevated in Aβ-treated cells	miRNA107	*In-vitro* mediation of cell viability, proliferation, inflammation, and apoptosis.	Negative regulation of FGF7/FGFR2/PI3K/Akt signal pathway	Chen et al., 2020

*NGF, nerve growth factor; tPA, tissue plasminogen activator; Aβ, amyloid beta PAI-1, plasminogen activator inhibitor 1; uPA, urokinase plasminogen activator; LRP-1, Low density lipoprotein receptor-related protein 1; MMPs, Matrix metalloproteinases; JAK/STAT, Janus kinase/signal transducer and activator of transcription; p-JAK2, phosphorylated Janus kinase 2; p-STAT3, phosphorylated signal transducer and activator of transcription 3; NF-κB, nuclear factor kappa B; FGF7, Fibroblast growth factor7.*

## Vascular Dysfunction and Cognitive Impairment

Recently, there has been an increasing interest in the relationship between vascular dysfunction and dementia. Clinicopathological and epidemiological studies have shown that various forms of systemic vascular disorders and cerebrovascular disease contribute to cognitive impairment and dementia (He et al., 2020). Several modifiable vascular risk factors are linked to AD type of dementia. These include hypertension, diabetes mellitus, metabolic syndrome, hypercholesterolemia, obesity, and smoking (Akinyemi et al., 2013). Vascular risk factors are associated with higher tau burden (Vemuri et al., 2017), higher cerebral beta-amyloid (Aβ) deposition (Langbaum et al., 2012; Gottesman et al., 2017), and act with Aβ synergistically to stimulate cognitive decline (Rabin et al., 2018).

Vascular dysfunction is a significant early component of AD pathology (Sweeney et al., 2019). The brains of AD patients are usually marked by abnormal NFT and amyloid plaque deposition not just in and around the neurons, but also in the blood vessels and perivascular space as well (Merlini et al., 2016). This leads to morphological changes and dysfunction of the vascular wall components (Greenberg et al., 2004). The tau, amyloid, and vascular dysfunction collectively contributed toward a speedy cognitive decline (Merlini et al., 2016).

The dense network of macro- and microvasculature supplies the brain with a continuous but regulated cerebral blood flow (CBF). The neurovascular unit (NVU) describes a structural and functional relationship among the cerebral microvascular cells (endothelium, pericytes and adventitial cells), neurons, and glial cells (astrocytes, microglia and oligodendrocytes). The NVU ensures a coherent response to injury to any components of the unit that are vulnerable to amyloid (Iadecola, 2004). Cerebrovascular autoregulation and the BBB are essential to sustain the NVU’s integrity (Bell and Zlokovic, 2009).

Cerebrovascular autoregulation maintains the CBF despite the changes in mean arterial pressure, while the BBB restricts the entry of toxic substances into the brain through its impermeable membrane (Bell and Zlokovic, 2009). Endothelial dysfunction associated with vascular risk factors such as hypertension and diabetes mellitus may cause the breakdown of these defense mechanisms, leading to BBB leakage, loss of autoregulation, cerebral hypoperfusion, and hypoxia (Kivipelto et al., 2002; Solomon et al., 2009). Additionally, Aβ accumulation within the microvessels itself further aggravates the endothelial damage (Jeynes and Provias, 2006; Burgmans et al., 2013). Brain magnetic resonance imaging of patients with clinically diagnosed AD often showed white matter changes demonstrated as white matter hyperintensities (WMH). WMH corresponds to areas of reduced CBF and hypoxia (Yamaji et al., 1997; Takahashi et al., 2000).

Neuroimaging has emerged as a revolutionary method to capture the alteration in CBF and its link to cognitive impairment and AD. Among the neuroimaging techniques to measure CBF include but are not restricted to functional MRI, single-photon emission computed tomography, arterial spin-labeling MRI, and dynamic susceptibility contrast MRI (de la Torre, 2018). A prospective study using cerebral arterial spin-labeling-MRI (ASL) in cognitively intact elderly subjects showed that compared to subjects who did not deteriorate to MCI, subjects who developed MCI 18 months later had a global reduction in CBF as captured during baseline ASL (Xekardaki et al., 2015). This study shows that ASL is a useful neuroimaging method to predict the onset of MCI in cognitively intact individuals whose brains showed abnormal CBF. Nevertheless, decreased CBF has been persistently demonstrated in various brain regions before and during cognitive impairment and AD. The most affected brain regions are the hippocampus, posterior cingulate cortex, medial temporal lobe, and precuneus (Dai et al., 2009; Schuff et al., 2009; Alexopoulos et al., 2012; Park et al., 2012; Xekardaki et al., 2015). Since MCI is a high-risk state to progress into dementia, including AD, CBF assessment combined with neurocognitive tests can be used to find out the susceptibility of patients with MCI to convert to AD, thus allowing physicians to decide which patients need more vigorous intervention and close follow-up. The Alzheimer’s Disease Neuroimaging Initiative also recommended that neuroimaging for CBF should be appraised as the primary predictive biomarker for AD (Iturria-Medina et al., 2016).

Vascular disorders are strongly associated with disintegrated BBB and AD (Provias and Jeynes, 2014). The breach of the endothelial tight junctions that leads to intense vascular leakage at the BBB is a feature of the early stages of dementia and AD (Teri et al., 1997). The transport of Aβ across the BBB is typically regulated by two major transporter proteins, LDL-receptor-related protein-1 (LRP-1) and receptor for advanced glycation end products (RAGE). LRP-1 promotes the efflux of Aβ from the cerebral parenchyma to the vessels across the BBB (Deane et al., 2009). In contrast, RAGE facilitates Aβ influx from the circulation crossing the BBB into the cerebral parenchyma (Jeynes and Provias, 2008). An Aβ-RAGE interface enhances oxidative stress, neuroinflammation, and subsequent BBB disintegration, which further potentiates cerebral Aβ accumulation (Schmidt et al., 2009). Whereas P-glycoprotein (PGP) has been demonstrated to damper RAGE activity in brain capillary endothelial cells that compose the BBB (Candela et al., 2010). In short, an imbalance between LRP-1, RAGE, and PGP expression together with disintegrated BBB remarkably contributed to vascular dysfunction and AD.

Vasoactive regulatory proteins such as vascular endothelial growth factor (VEGF) and endothelial nitric oxide synthase (eNOS) also play a role in maintaining BBB integrity and therefore affect the cerebral Aβ transport (Farkas and Luiten, 2001; Jeynes and Provias, 2011). Patients with AD commonly have low serum VEGF levels (Mateo et al., 2007) and this is associated with progressive loss of cognitive function (Tang et al., 2013). Decreased expression of VEGF and eNOS within the cerebral microvasculature disrupts the normal angiogenesis and BBB integrity. This leads to less Aβ efflux across the BBB from the cerebral parenchyma into the circulation in the human brain with AD (Provias and Jeynes, 2014).

**FIGURE 2:**
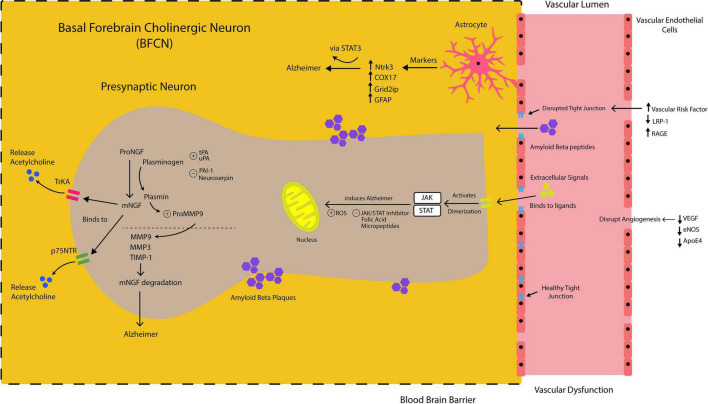
Nerve growth factor (NGF) metabolic pathway in basal forebrain cholinergic neuron (BFCN) is mainly regulated by proteases involved in NGF maturation [tissue plasminogen activator (tPA), urokinase plasminogen activator (uPA), Plasminogen Activator Inhibitor 1 (PAI-1) and Neuroserpin)] and those involved in NGF degradation [matrix metalloproteinases (MMP9, MMP3) and tissue inhibitor of metalloproteinases 1 (TIMP-1)]. Plasmin is also responsible for proMMP-9 activation to MMP9. Extracellular signals (i.e., cytokines, hormones, and growth factor) bind to their ligands and activate Janus Kinase/signal transducer and activator of transcription (JAK/STAT). This causes dimerization of the two and allows them to be translocated to the nucleus for transcriptional response. Reactive Oxygen Species (ROS) is involved in JAK/STAT-induced Alzheimer. JAK/STAT inhibitor, folic acid and micropeptides (i.e., humorin and colivelin) attenuate JAK/STAT-induced Alzheimer. Increased astrocytes biomarker Glial fibrillary acidic protein (GFAP) and Genes were predominantly expressed on Astrocytes- STAT3-induced Alzheimer’s disease (Neurotrophic Receptor Tyrosine Kinase 3 (ntrk3), Cytochrome C Oxidase Copper Chaperone (*COX17), and* Grid2 Interacting Protein (Grid2ip). Decreased vascular endothelial growth factor (VEGF), endothelial nitric oxide synthase (eNOS) and apolipoprotein e4 (ApoE4) disrupt normal angiogenesis. Increased vascular risk factor, receptor for advanced glycation end products (RAGE) and reduced LDL-receptor related protein-1 (LRP-1) disrupt healthy tight junction. Disruption of normal angiogenesis and healthy tight junction allow Amyloid-Beta peptides efflux from vessels to brain parenchyma and promoting Amyloid-Beta plaques formation.

Genome-wide association studies have revealed a number of genetic associations with AD (Naj et al., 2017), of which the most important is the apolipoprotein E gene on chromosome 17 (APOE gene, apoE protein) (Apostolova et al., 2018). Other polymorphisms are associated with genes linked to lipid metabolism, inflammation, and intracellular trafficking, but none of them are as consistent as that of apoE (Bis et al., 2020). ApoE is a component of very-low-density lipoproteins that is involved in cholesterol transport among various cells in the body. ApoE4 allele frequency is higher in patients with vascular dementia, AD, and ischemic cerebrovascular disease (Ji et al., 1998). ApoE4 carriers with atherosclerosis commonly have co-morbid AD, cognitive impairment, and dementia (Slooter et al., 1999). AD patients who are ApoE4 carriers have a higher Aβ burden compared with non-carriers (Dorey et al., 2014). ApoE4 disrupts brain lipid metabolism, impedes CBF, and triggers cerebral amyloid angiopathy (Reinvang et al., 2013). The binding of Aβ to ApoE reduced LRP-1 functioning in mice (Bell et al., 2007). Furthermore, an *in vitro* BBB model comprising mouse brain capillary endothelial cells demonstrated that ApoE4 disrupts endothelial tight junction integrity and BBB permeability (Nishitsuji et al., 2011; Figure 2).

## Omics in Alzheimer’s Disease

Fresh molecular and high-throughput methods are shedding new insight into the pathways and networks at the heart of this complicated disease. This is required in order to go forward with the precision medicine paradigm toward the discovery of novel molecular markers, systematic risk categorization, and translational targeted medicines (Kosik, 2015; Castrillo and Oliver, 2016; Kovacs, 2016).

The implementation of a complete precision medicine strategy, as well as the creation of relevant biomarkers, are likely to result in significant advancements in recognizing, treating, and preventing AD.

AD and many other genetically heterogeneous illnesses (e.g., autism, other NDs, and some malignancies) are far too varied to be the sole cause or to be centered on genetic variants. Certain complicated disorders are distinguished by the following characteristics: (1) multifactorial in nature, integrating genetic, epigenomic, and interactomics factors; (2) primarily caused by “modified” networks that influence important modules and interactomes; and (3) deeply complicated, with a strong interplay between impacted and homeostatic defense systems. As a result, for many complex disorders, comprehensive system-level therapies are necessary (Castrillo and Oliver, 2016).

Advanced theoretical and statistical systems biology techniques are being applied to better understand the pathophysiology of multifactorial illnesses such as neurodegenerative illnesses. These methods include high-throughput omics techniques that investigate genome, transcriptome, proteome, and metabolome patterns and interactions, as well as integrative approaches (Castrillo and Oliver, 2016). Multi-omics trials are currently being utilized in integrated personal omics profiling (iPOP) longitudinal research to track panels of biomarkers and trends for diagnosis and customized therapy (Chen et al., 2012).

Among the most important systems-level techniques, including the use of omics technology to dissect AD (Tosto and Reitz, 2016), are 1. Transcriptomics: RNA-Seq to elucidate early patterns of dysregulation underlying ADe (Chen et al., 2016); experimental approaches applied to the analysis of RNA networks in AD (Pichler et al., 2017); RNA-Seq experiments demonstrating (Bai et al., 2014), 2. Proteomics investigations indicate that amyloid disrupts splicing pathways (Nuzzo et al., 2017), metalloproteomics and redox proteomics studies in AD (Hare et al., 2016), and plasma proteomics biomarkers in AD (Perneczky and Guo, 2016), 3. Metabolomics: AD-related changes in metabolic processes and networks (Toledo et al., 2017).

The human blood metabolome is made up of hundreds of tiny molecular species, most of which have a molecular weight of less than 1,500 Da (Daltons; 1.7 1027 kg) and are predominantly composed of monosaccharides, acylcarnitines, biogenic amines, amino acids, fatty acids, and complex lipids. The metabolome is regarded as a downstream linear mirror of the genome/epigenome, transcriptome, and proteome, sequentially and in close proximity to the clinical phenotype, using typical reductionistic methodologies. Although the aforementioned may be accurate from a systems biology standpoint, intricate interrelationships occur between the many “omic” levels (Yugi et al., 2016), which, if correctly integrated, are likely to give a greater understanding of a complicated disease state or human health.

Recent reports of metabolomic biomarkers for AD have been developed using specimens from cross-sectional studies analyzed with MS platforms, typically comparing metabolite abundances between control subjects and individuals with either prodromal or manifest AD (Klavins et al., 2015).

Recent approaches in the treatment of AD include exploring the potentials of some metabolites to modulate signaling pathways associated with neurovascular endothelium through multi-omic analyses (Corral-Jara et al., 2021). There have also been reports on cellular signaling-related sex-dependent effects under hyperglycemic and lipid stress (Nuthikattu et al., 2020, 2021). However, this review focuses on significant signaling pathways associated with the stages of AD, the potential links between vascular dysfunction and AD, as well as recent developments in “omics”-based approaches in AD. Without publication date restriction, PubMed, ScienceDirect, and Google Scholar databases were searched for published articles containing the search terms related to “Alzheimer,” “dementia,” “signaling pathways,” “vascular dysfunction,” “cognitive impairment,” and “omics.”

## Summary and Future Directions

In this review, we discussed many signaling pathways and postulated mechanisms associated with AD. Mild cognitive impairment (MCI) can be an early stage of the disease continuum for dementia, including AD. In line with this, the aforementioned signaling markers are being increasingly studied as potential early diagnostic or prognostic markers for cognitive decline-related pathologies. As for the NGF pathway, a meta-analysis and systematic review of 170 studies reported a significant increase in the peripheral levels of NGF along with other proinflammatory markers in MCI compared with controls (Shen et al., 2019). More recently, markers associated with the NGF pathway, such as proNGF, plasminogen, neuroserpin, and MMP9 was shown to be elevated, whereas mNGF was downregulated in the brain tissue of MCI and AD patients (Pentz et al., 2020). In line with this, Senescence-Associated Secretory Phenotype (SASP) biomarkers, which is upstream of JAK/STAT signaling at the cellular level, has been reported to be elevated in the plasma of older adults with MCI, MCI + Major Depressive Disorder (MDD), and MDD (Diniz et al., 2021). To date no reports have directly implicated FGF7/FGFR2 as early markers of cognitive impairment at the clinical level, hence more studies should look into this.

It is important to distinguish between brain aging and AD dementia. With age, our brains experience natural structural, chemical and functional degenarations, as well as significant cognitive decline, which is characterized by brain shrinkage, decreased blood flow, synapse degeneration, and neurochemical alternations (Damoiseaux, 2017). Most of the human studies, on the other hand, seldom exclude prevalent health disorders associated with aging, such as hypertension, which may have an impact on how accurate the conclusions are. The study of age-related brain alterations in non-human animal models is therefore essential for distinguishing between normal aging and pathological brain abnormalities in humans. Studying the basic processes of aging in animals will consequently provide an essential foundation for the development of innovative therapies for age-related brain problems in people (Alexander et al., 2020).

Normal aging has been shown to be associated with significant changes in the brain network and the processes that it controls (Huang et al., 2015; Bonte et al., 2017). The human brain’s network has been reorganized as a result of aging-related changes in functional, metabolic, and structural connections, according to recent research. It has been discovered that functional connection reduces with age, both within and between numerous resting-state networks, including the default mode network, salience network, dorsal attention network, and sensorimotor network (Huang et al., 2015). The aged also have lower modularity and efficiency in their brain networks (Liu et al., 2014; Geerligs et al., 2015; Zhao et al., 2015), as well as a degeneration process in which the aging brain system transitions from a small-worldness network to a regular network in conjunction with normal aging. The findings of functional connectivity studies (Damoiseaux, 2017) show that age-related changes in structural network metrics are comparable to those seen in structural network metrics.

A good method for evaluating energy consumption in neurons is [18F] fluorodeoxyglucose positron emission tomography (18F-FDG PET), which is a reflection of neural transmission signals (Rocher et al., 2003). As a result, 18F-FDG PET is regarded as a functional neuroimaging tool for identifying changes in brain activity associated with advancing years of age. A large number of studies have been conducted using 18F-FDG PET to explore the changes in brain processes that occur as a result of normal aging throughout the past several decades. Overall, a considerable age-related reduction in glucose metabolism in the frontal lobe has been seen in most investigations (Pardo et al., 2007; Kalpouzos et al., 2009), which is consistent with other findings. Glucose uptake may be decreased as a result of tissue loss or shrinking (Bonte et al., 2017). As well as in metabolic brain networks, age-related alterations can be observed in association and paralimbic cortex areas, including increased clustering, decreased efficiency and robustness, as well as altered nodal centralities (Rocher et al., 2003; Liu et al., 2014). Whether the age-related changes in glucose metabolism are consistent between the rat brain and the human brain, particularly in terms of metabolic brain networks, has not been determined conclusively at this point. It is yet unknown if the metabolic network in the brains of old rats exhibits age-related alterations that are comparable to those observed in the brains of humans.

A recent study of single-cell transcriptome datasets from the human brain revealed a link between AD gene signals and microglia, as well as astrocytes and astrocyte-like cells. Studies of human microglia (MG) produced from embryonic stem cells in the mouse brain have shown the cells are transcriptionally like human primary microglia ex vivo and express genes associated with Alzheimer’s disease (AD). However, just one-third of the suspected Alzheimer’s disease risk genes have appropriate mouse orthologs (Mancuso et al., 2019), which is concerning. Furthermore, oligomeric amyloid-beta causes a different response in human microglia compared to mice microglia (MG). As research advances, it is becoming increasingly clear that signals from the CNS microenvironment are required to maintain microglial specification, and that the absence of these cues results in a dramatic disruption of the microglia phenotype, driving them toward an activated state. Additionally, certain well-known disease-associated genes (for example, triggering receptor expressed on myeloid cells 2 (TREM2)–membrane phospholipids–apolipoprotein E (APOE), CD33–sialic acid) play a role in the cross-talk between microglia and other brain cells.

Soreq et al. (2017) recently discovered significant differences in glial gene expression as well as neuronal and oligodendrocyte (OLG) cell counts in the brains of elderly people as compared to young people. A total of 1,231 postmortem brain samples from 134 people (ages 16–104), profiled by exon microarrays (10 brain areas), and 480 samples from the North American brain bank were analyzed (Soreq et al., 2017; Elmore et al., 2018). The 3′ microarray data from microglia-depleted animals (including comparisons between young and elderly mice) was also used to analyze the mice. Authors discovered considerable alterations in gene expression. Similarly, significant changes in AD may be observed in the future.

A detailed analysis of RNA-Seq data can be performed using machine learning and statistics, including classification methods [for example, unsupervised hierarchical classification (HCL) and bioinformatic algorithms], correlation analyses, and the detection of gene expression changes in cell-specific markers, pro-inflammatory genes, and splicing factors, as well as genes associated with the cell cycle. Linear regression may also be used to identify genes that have been changed. Functional Gene Ontology (GO) analysis, which is non-parametric, may detect changes in molecular functions (MF) and biological processes (BP) (Luo and Chen, 2016). The genes that were discovered can also be examined in the human cell Atlas (Regev et al., 2017). Aside from that, developments in genetics and sequencing have uncovered an abundance of disease-associated genetic modifications; nonetheless, precise models for molecular dissection are still needed to fully understand these changes.

The precision of molecular medicine is significantly improved by using a multi-omics approach. When it comes to precisely understanding the impact of mutations or therapies, single-cell studies are crucial. Transcriptional networks may be constructed from single-cell data, if necessary. The information obtained may aid in gaining a better understanding of the impact of altered genes on disease pathophysiology. Cell lines or model organisms can be used to determine the underlying cause of disease, but other technologies are required to complete the task. The introduction of single-cell RNA-Seq had a significant influence on the field. It is possible to obtain accurate cell state comparisons by comparing healthy samples to sick samples in the same experiment. Major faults may be identified *via* cell harmony analysis, and genetic rescue of mutations is also conceivable after investigations of cell state models and more clinical Alzheimer’s disease samples. Additionally, differentiating gene expression across distinct cell states may be investigated.

When analyzing data, clustering is a way of grouping closely related observational findings into groupings called “clusters” that are based on the results of numerous variables for each subject. Because it serves as a critical intermediatory tool in experimental investigations, it is incredibly significant in scientific research. However, it is particularly valuable in gene analysis because it contributes to the knowledge of specific cell processes. This easy and helpful adaptation to a variety of different scientific fields has not only enabled its widespread usage but has also facilitated the creation of a variety of distinct clustering methodologies. Because of this, these sophisticated approaches may be utilized to struttingly analyze data in order to give trustworthy gene analysis (Park et al., 2009; Habib et al., 2020). Other research has discovered that previously illness-associated astrocytes are changed in AD and aging using single-cell RNA-Seq, demonstrating that astrocytes are linked to hereditary and age-related disease factors (Habib et al., 2020).

## Conclusion

The pathogenesis of AD is multifactorial. Continuous discovery of novel signaling pathways in AD reflects the complex nature of the disease. Such complexity should be addressed in patients’ management for better treatment outcomes, and tools such as omics are better fit to be incorporated into treatment plans. Vascular dysfunction has a deleterious impact on the brain that builds up to AD pathogenesis. Since measures to tackle the vascular risk factors are available, the future prevalence of AD can be minimized by employing strategies that preserve cardiovascular health.

## Author Contributions

JK contributed to the conceptual framework and design of the manuscript. MA, KS, AU, WM, HK, NHI, and JK drafted the manuscript. HK contributed to the preparation of figures and figure legends. MA, KS, AU, WM, CK, and JK critically revised the manuscript. All authors contributed to the article and approved the submitted version.

## Conflict of Interest

The authors declare that the research was conducted in the absence of any commercial or financial relationships that could be construed as a potential conflict of interest.

## Publisher’s Note

All claims expressed in this article are solely those of the authors and do not necessarily represent those of their affiliated organizations, or those of the publisher, the editors and the reviewers. Any product that may be evaluated in this article, or claim that may be made by its manufacturer, is not guaranteed or endorsed by the publisher.

## Footnotes

1
http://alzpathway.org/

## Glosarry

AD, Alzheimer’s disease; COVID-19, corona virus disease-2019; HIV, human immunodeficiency virus; APP, amyloid precursor protein; Aβ, amyloid beta; MAPT, microtubule-associated protein tau; ApoE, apolipoprotein E; NFT, neurofibrillary tangles; Wnt, Wingless-related integration site; MAPK, mitogen-activated protein kinase;NFTs, neurofibrillary tangles; NMDARs, *N*-methyl-d-aspartic acid receptors; NGF, Nerve growth factor; PNS, peripheral nervous system; CNS, central nervous system; BFCN, basal forebrain cholinergic neurons; proNGF, precursor nerve growth factor; mNGF, mature nerve growth factor; TrkA, tropomyosin-related kinase A; p75NTR, p75 neurotrophin receptor; tPA, tissue plasminogen activator; uPA, urokinase plasminogen activator; PAI-1, plasminogen activator inhibitor 1; MMPs, matrix metalloproteinases; MMP-9, matrix metalloproteinases; TIMP-1, tissue inhibitor of metalloproteinases 1; proMMP-9, precursor matrix metalloproteinases-9; MMP-3, matrix metalloproteinases-3; MMP-1, matrix metalloproteinases-1; MMP-2, matrix metalloproteinases-2; MMP-13, matrix metalloproteinases-13; MT1-MMP, Membrane type 1 matrix metalloproteinase; JAK/STAT, Janus kinase/signal transducer and activator of the transcription; JAK1, Janus kinase 1; JAK2, Janus kinase 2; JAK3, Janus kinase 3; Tyk2, Tyrosine kinase 2; STAT1, signal transducer and activator of the transcription 1; STAT2, signal transducer and activator of the transcription 2; STAT3, signal transducer and activator of the transcription 3; STAT4, signal transducer and activator of the transcription 4; STAT5A, signal transducer and activator of the transcription5A; STAT5B, signal transducer and activator of the transcription 5B; and STAT6, signal transducer and activator of the transcription 6; Nrf2, nuclear erythroid 2-related factor 2; TREM2, triggering receptor expressed on myeloid cells 2; Ntrk3 neurotrophic tyrosine kinase receptor type 3; Cox17, Cytochrome C Oxidase Copper Chaperone; Grid2ip, Grid2 Interacting Protein; TGF-β, transforming growth factor-beta; GFP, glial fibrillary acidic protein; EAATs, excitatory amino acid transporters; ALDH1L1, aldehyde dehydrogenase 1 family member L1; FGFs, Fibroblast growth factors; FGFRs, Fibroblast growth factor receptors; AKT, Protein kinase B; P13K, phosphatidylinositol 3-kinases; GRB2, Growth factor receptor-bound protein 2; GAB1; GRB2 Associated Binding Protein 1; CSF, cerebrospinal fluid; FGF7, Fibroblast Growth Factor 7; mir-107, MicroRNA-107.
